# The G Allele of CaSR R990G Polymorphism Increases Susceptibility to Urolithiasis and Hypercalciuria: Evidences from a Comprehensive Meta-Analysis

**DOI:** 10.1155/2015/958207

**Published:** 2015-02-01

**Authors:** Kang Liu, Xiaolan Wang, Jiaxin Ye, Chao Qin, Pengfei Shao, Wei Zhang, Jie Li, Changjun Yin

**Affiliations:** Department of Urology, The First Affiliated Hospital of Nanjing Medical University, Nanjing 210029, China

## Abstract

*Background*. The calcium-sensing receptor gene (CaSR) is a candidate to explain urolithiasis. A number of case-control studies were conducted to investigate associations between CaSR polymorphisms with risks of hypercalciuria and urolithiasis in humans. But the results were still inconsistent. *Methods*. A meta-analysis was performed to address this issue. Crude odds ratios (ORs) with 95% confidence intervals (CIs) were calculated to estimate the strength of associations between CaSR polymorphisms and the risk of urolithiasis. The pooled standardized mean difference (SMD) with 95% CI was used for the meta-analysis of CaSR polymorphisms and urine calcium concentration. *Results*. For urolithiasis association, the SS genotype of A986S polymorphism was a risk factor for urolithiasis in Asians and PHPT patients, but a protective factor in Caucasians. The GG genotype of R990 polymorphism was associated with an increased risk of urolithiasis, especially in Caucasians and healthy population. Regarding urine calcium concentration association, individuals with the G allele had a higher level of urine calcium than the noncarriers. *Conclusions*. This meta-analysis revealed that the G allele of CaSR R990G polymorphism increases susceptibility to urolithiasis and hypercalciuria. The A986S and Q1011E polymorphisms were associated with urolithiasis and hypercalciuria in specific populations.

## 1. Introduction

Urolithiasis is a condition that has been recognized for centuries and is the third most common cause of urinary tract disease [1]. Nearly 5% of females and 12% of males are likely to develop urolithiasis during their lifetime [2]. Urolithiasis is a global health problem with a 40 to 50% recurrence rate within five years [3]. In recent years, many studies have made utmost efforts to investigate the pathogenesis of urolithiasis. However, the detailed pathogenic mechanism for the occurrence and recurrence of urolithiasis remains unknown.

Urolithiasis is a multifactorial disease which is considered to be associated with the effects of multiple genes in combination with lifestyles and environmental influences [4]. Although no specific gene has been declared to be the underlying cause of urolithiasis, many functional genes such as urokinase, vitamin D receptor gene (VDR), and calcium-sensing receptor gene (CaSR) have been verified to be related to urolithiasis [5–7]. The CaSR gene, located on chromosome 3q13.3-21, spans 103 kb and encodes for a protein of 1078 amino acids present in the plasma membrane. CaSR is a member of the G-protein coupled receptors and its structure has 3 different domains [8, 9]. It is widely accepted that CaSR may be connected with urolithiasis, since it decreases calcium reabsorption in thick ascending limbs and distal convoluted tubules, increases phosphate reabsorption in proximal tubules, and decreases water and proton reabsorption in collecting ducts [10]. As a consequence, the CaSR gene is a candidate to explain the susceptibility to urolithiasis.

The CaSR gene is composed of seven exons, the first six coding for the extracellular domain of the CaSR protein and exon 7 coding for the transmembrane and the intracellular domains [11]. Three single-nucleotide polymorphisms (SNPs), A986S (rs1801725, G > T), R990G (rs1042636, A > G), and Q1011E (rs1801726, C > G), located on exon 7, are extensively studied [12]. Shakhssalim and his colleagues observed a significantly higher frequency of the 986S, 990G, and 1011Q alleles in stone formers [13]. Another Italian study puts forward similar conclusions [14]. These findings confirm that CaSR gene polymorphisms may be involved in urolithiasis, but the impact of these amino acid changes on the function of CASR is not well defined. Unfortunately, we have no sufficient knowledge to resolve these puzzles.

At present, several studies have attempted to investigate associations between CaSR gene variants with urolithiasis and urinary calcium concentration. However, the results were inconsistent or even contradictory. To date, no one has conducted a meta-analysis to further probe the associations. To fill this gap, we performed a meta-analysis of all eligible studies to derive more reliable estimation of associations between calcium-sensing receptor gene polymorphisms with urolithiasis and urinary calcium concentration.

## 2. Materials and Methods

### 2.1. Identification of Eligible Studies

A comprehensive literature search was performed through the PubMed, Medline, Embase, and Web of Science databases for relevant articles published (the last search update was June. 30, 2014) with the following key words: “CaSR,” “polymorphism,” “variation,” or “mutation,” and “urolithiasis,” or “calculi” and in combination with “urine calcium excretion.” Additional studies were identified by hand, searching references in original articles and review articles.

### 2.2. Inclusion and Exclusion Criteria

The included studies needed to meet the following criteria. (1) The study examined the associations between CaSR polymorphisms and urinary calcium concentration and/or urolithiasis risk. (2) For urolithiasis association, the study must be case-control study and must have clear original data of genotypic and allelic frequencies. (3) For urinary calcium concentration association, the study must have clear original data of the mean of urinary calcium concentration and standard deviations (SD) by genotypes. In addition, the number of each genotype must be clear. Major reasons for exclusion of studies were as follows: (1) not for urolithiasis or urinary calcium concentration research, (2) review articles, (3) only case population, and (4) duplicate of previous publication.

### 2.3. Data Extraction

Information was carefully extracted from all eligible studies independently by two investigators according to the inclusion criteria listed above. For conflicting evaluation, a consensus was reached by discussion. The following information was collected from each study: the first author's name, the year of publication, ethnicity, country of origin, genotyping method, source of control groups (population- or hospital-based controls), subjects, numbers of cases and controls, frequency of CaSR polymorphisms in cases and controls, the mean of urinary calcium concentration, and SD by genotypes. Meanwhile, the corresponding and first authors of the published studies were contacted by sending e-mails if they did not provide their original data.

### 2.4. Statistical Analysis

Crude odds ratios (ORs) with their corresponding 95% CIs were used to assess the strength of associations between CaSR polymorphisms and urolithiasis risk. The pooled ORs were performed for homozygote model (M/M versus W/W), heterozygote model (W/M versus W/W), dominant model (W/M + M/M versus W/W), and recessive model (M/M versus W/W + W/M), respectively. Between-study heterogeneity was checked by the chi-square-based *Q* test (heterogeneity was considered statistically significant if *P* < 0.10) [24]. Pooled OR estimate of each study was calculated by both the fixed-effects model (the Mantel-Haenszel method) and the random-effects model (the DerSimonian and Laird methods). The fixed-effects model would be adopted when the studies were found to be homogeneous (with *P* > 0.10 for the *Q* test). Otherwise, the random-effects model would be applied. To better investigate the possible sources of between-study heterogeneity, meta-regression analysis was performed. In addition to the comparison among all subjects, we also conducted stratification analyses by ethnicity, subjects, and source of controls. Sensitivity analyses were performed to assess the stability of the results; namely, a single study in the meta-analysis was deleted each time to reflect the influence of the individual data set to the pooled OR. The pooled standardized mean difference (SMD) with 95% CI was used for the meta-analysis of CaSR polymorphisms and urinary calcium concentration. Begg's funnel plot and Egger's linear regression test were used to assess publication bias. Moreover, departure from Hardy-Weinberg equilibrium (HWE) in controls was tested by the chi-square test for goodness of fit, and a *P* < 0.05 was considered as a significant disequilibrium. All statistical analyses were performed with the Stata software (version 12.1; StataCorp LP, College Station, TX, USA), using two-sided *P* values.

## 3. Results

### 3.1. Characteristics of Studies

There were 43 articles relevant to the search words, of which 34 articles were excluded. Of the 34 excluded studies, 11 articles were not related to A986S, R990G, and Q1011E polymorphisms, 8 studies were review articles, and 15 papers were not case-control studies. Besides, 3 additional articles [17, 20, 21] were identified by hand. Twelve papers were finally included in this meta-analysis.

**Figure 1:**
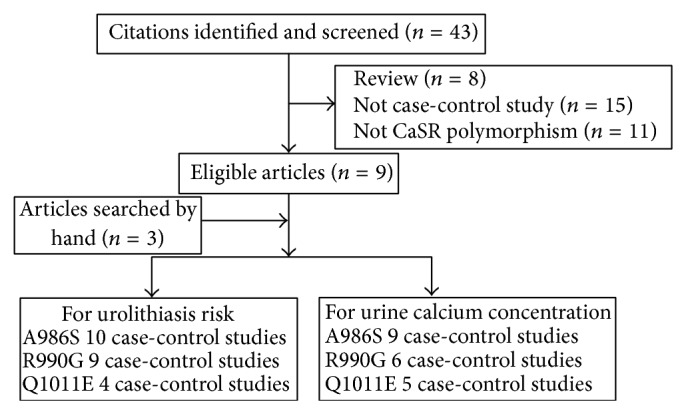
Studies identified with criteria for inclusion and exclusion.

For the associations between CaSR polymorphisms and urolithiasis, a total of seven papers which included 10 case-control studies [5, 13–18] for the A986S polymorphism, 9 case-control studies [13–18] for the R990G polymorphism, and 4 case-control [13, 14, 17] studies for the Q1011E polymorphism were involved in this meta-analysis.  Figure 1 graphically illustrates the trial flow chart. All studies used blood samples for DNA extraction, while polymerase chain reaction method, TaqMan, or DNA sequencing methods were used for genotyping. Controls were mainly matched on sex and age. In addition, the distribution of genotypes in the controls of all studies was consistent with Hardy-Weinberg equilibrium. Main characteristics for all case-control studies were listed in Table 1.

For the associations between CaSR polymorphisms and urinary calcium concentration, ten papers [5, 13–15, 18–23] were retrieved according to the inclusion criteria. The process of study selection is also shown in Figure 1. Hereinto, nine (1670 individuals), six (1049 individuals), and five (964 individuals) studies were included in the meta-analysis for the association between A986S, R990G, and Q1011E polymorphisms and urinary calcium concentration, respectively.  Table 2 listed the characteristics of these studies.

### 3.2. Association between CaSR Polymorphisms and Urolithiasis Risk

**Figure 2:**
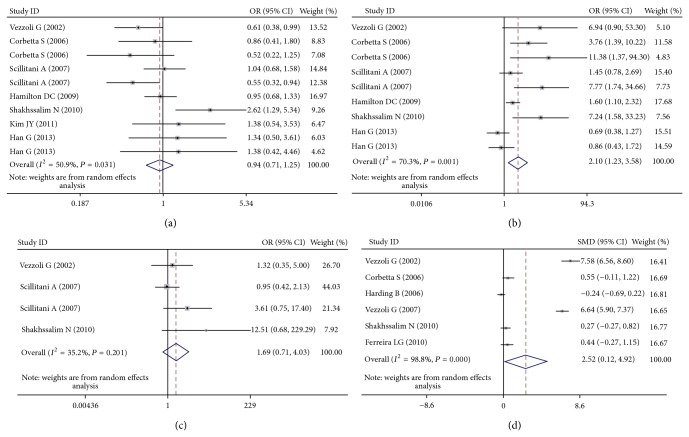
(a) Forest plot of urolithiasis risk associated with the CaSR A986S polymorphism under the dominant model. (b) Forest plot of urolithiasis risk associated with the CaSR R990G polymorphism under the dominant model. (c) Forest plot of urolithiasis risk associated with the CaSR Q1011E polymorphism under the dominant model. (d) Forest plot of urine calcium concentration associated with the CaSR R990G polymorphism under the dominant model.

**Figure 3:**
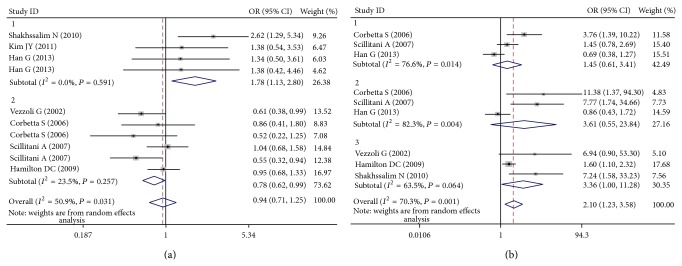
(a) Forest plot of the CaSR A986S polymorphism associated with urolithiasis risk stratified by ethnicity (AS + SS versus AA). (b) Forest plot of the CaSR R990G polymorphism associated with urolithiasis risk stratified by subjects (RG + GG versus RR).

**Figure 4:**
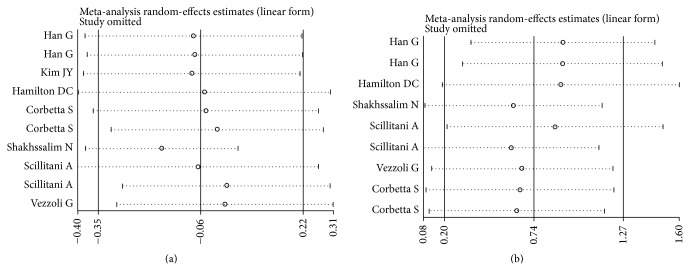
(a) Sensitivity analysis of urolithiasis risk associated with the CaSR A986S polymorphism under the dominant model. (b) Sensitivity analysis of urolithiasis risk associated with the CaSR R990G polymorphism under the dominant model.

**Figure 5:**
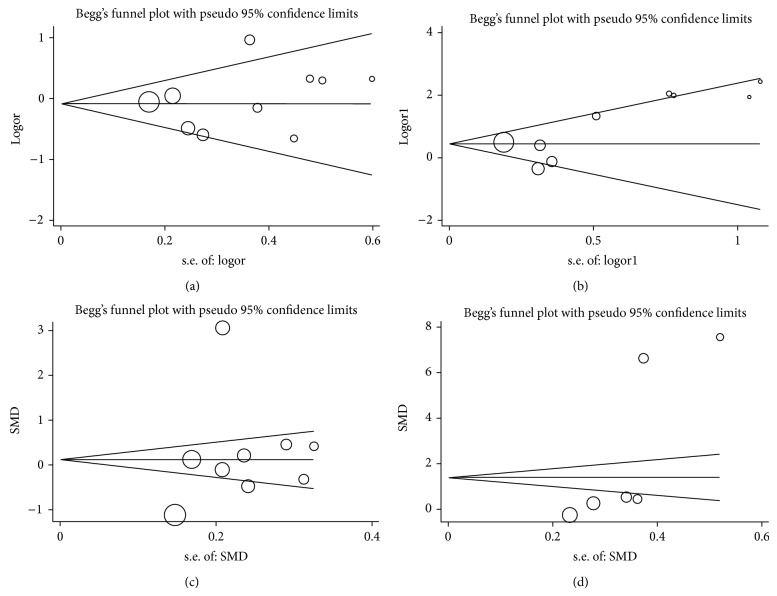
(a) Begg's funnel plot for publication bias test of urolithiasis risk associated with the CaSR A986S polymorphism. (b) Begg's funnel plot for publication bias test of urolithiasis risk associated with the CaSR R990G polymorphism. (c) Begg's funnel plot for publication bias test of urine calcium concentration associated with the CaSR A986S polymorphism. (d) Begg's funnel plot for publication bias test of urine calcium concentration associated with the CaSR R990G polymorphism.

Table 3(a) lists the main results of the meta-analysis of the associations between A986S polymorphism and urolithiasis risk. Overall, no obvious association was observed in all the genetic models (Figure 2(a)). However, there was noteworthy heterogeneity between studies. Hence, we then performed subgroup analysis. Through stratified analyses, the heterogeneity of the subgroup notably reduced. In the subgroup analysis on ethnicity, we discovered that the A986S polymorphism was significantly associated with an increased urolithiasis risk in Asians under the heterozygote model (OR = 1.64, 95% CI = 1.04–2.56) and the dominant model (OR = 1.78, 95% CI = 1.13–2.89; Figure 3(a)). Interestingly, a conflicting association was found in Caucasians under the dominant model (OR = 0.78, 95% CI = 0.62–0.99). Stratified analysis was also performed by subjects. The results indicated that primary hyperparathyroidism (PHPT) patients with the A/A genotypes had a significantly lower urolithiasis risk in the dominant model (OR = 0.62, 95% CI = 0.40–0.96). In the stratified analysis by source of controls, significant associations were found in hospital-based group (dominant model: OR = 0.67, 95% CI = 0.46–0.96). We next conducted a leave-one-out sensitivity analysis to determine whether a particular study or studies would result in heterogeneity. Finally, the omission of individual studies did not materially alter the results (Figure 4(a)). The sensitivity analysis thus confirmed that the results were statistically robust. In addition, as shown in Figure 5(a), no possibility of publication bias for this test was observed.

The relationship between the R990G polymorphism and the risk of urolithiasis is summarized in Table 3(b). Significant associations were observed in the dominant genetic model (OR = 2.10, 95% CI = 1.23–3.58; Figure 2(b)). In the stratified analysis by ethnicity, the positive results were found only in the Caucasian subgroups, but not in the Asian populations. The pooled OR was 2.17 (95% CI = 1.50–4.91) in Caucasian subgroups for the dominant model. When the studies were stratified by subjects, healthy individuals with the R/R genotypes were related to a significantly decreased risk of urolithiasis (dominant model: OR = 3.36, 95% CI = 1.00–11.28; Figure 3(b)). Moreover, we failed to find any effects on urolithiasis risk in all genetic models tested when restricting the analysis to the source of controls. It is worth noting that the heterogeneity remained significant after subgroup analysis. Therefore, we used meta-regression analysis to explore the source of heterogeneity by ethnicity, subjects, source of controls, and year of publication. We found that only the year of publication contributed to substantial altered heterogeneity, which could account for 100% source of heterogeneity. Moreover, the sensitivity analysis was also conducted (Figure 4(b)). After individual study omission, the corresponding pooled OR was not altered significantly. Begg's funnel plot and Egger's test did not suggest evidence of publication bias (Figure 5(b)).

As shown in Table 3(c), we did not observe any significant associations between the Q1011E polymorphism and urolithiasis risk in all the genetic models (Figure 2(c)). The Q1011E variant has been reported to be much less frequent than the other two SNPs. Given heterogeneity was not remarkable in all the genetic models and only four case-control studies were finally included, we did not conduct subgroup analysis. The sensitivity analysis showed that results were reliable and stable. Besides, Begg's funnel plot and Egger's test were performed to evaluate the publication bias of the literatures. Similarly, no publication bias was detected for association of Q1011E polymorphism with urolithiasis.

### 3.3. Association between CaSR Polymorphisms and Urinary Calcium Concentration

The results of the overall meta-analysis provided a strong evidence of the association between urinary calcium concentration and the CaSR R990G polymorphism (SMD = 2.52, 95% CI: 0.12–4.92, and *P* = 0.039; Figure 2(d)), but not the A986S polymorphism (SMD = 0.25, 95% CI: −0.59–1.10, and *P* = 0.56) and the Q1011E polymorphism (SMD = −1.19, 95% CI: −2.52-0.15, and *P* = 0.081). Furthermore, we performed subgroup analysis (Table 4). The stratified analysis only showed that Caucasians with the Q1011E polymorphism ancestral genotype had significantly higher urinary calcium concentration than those with the minor allele (SMD = −1.99, 95% CI: −3.74 to −0.24, and *P* = 0.026). Unfortunately, we did not obtain other positive findings.

There was heterogeneity among studies in overall comparisons. To explore the sources of heterogeneity, we conducted subgroup analyses by ethnicity and subjects. However, the heterogeneity remained significant. Egger's test and Begg's funnel plot were applied for comparison to assess the publication bias of the literature, and no possibility of publication bias for this test was observed (A986S: Begg *P* = 0.466, Egger *P* = 0.493, Figure 5(c); R990: Begg *P* = 0.06, Egger *P* = 0.066, Figure 5(d); Q1011E: Begg *P* = 0.806, Egger *P* = 0.066).

## 4. Discussion

Prevalence of urolithiasis is epidemic in many regions of the world. It is acknowledged that urolithiasis is a major health problem, with a significant proportion of patients requiring extensive surgical procedure. However, it is still puzzling whether the increased risk of urolithiasis is attributable to genetic factors, environmental exposure, or some combination [4, 25]. In recent years, single nucleotide polymorphisms (SNP) have been identified as a powerful tool for predicting complex diseases [26]. As a candidate gene, calcium-sensing receptor gene has been widely noticed. Three missense polymorphisms of the CaSR gene (A986S, R990G, and Q1011E) have a significant frequency in general population [27]. Several investigators tried to examine associations between CaSR polymorphisms and urolithiasis risk, but the conclusions were conflicting. Meta-analysis is a powerful tool, which can provide more reliable results than a single study and explain controversial conclusions [28]. To our best knowledge, no one has conducted a meta-analysis to confirm the association between CaSR polymorphisms and urolithiasis. To fill this gap, we performed a meta-analysis of all eligible studies to derive more precise estimation. Finally, our meta-analysis indicated that the S allele of A986S polymorphism might be a risk factor for urolithiasis in Asians but be a protective factor in Caucasians. Besides, the G allele of R990G polymorphism might contribute a significant increased overall risk of urolithiasis, particularly in Caucasians and healthy populations. No significant associations were discovered in the Q1011E polymorphism.

A growing body of evidence shows that the CaSR might play a crucial role in the pathogenesis of urolithiasis. CaSR is a common protein which usually expressed in the parathyroid glands, renal tubules, and distal tubules [29]. CaSR is an important regulator of PTH secretion according to blood calcium concentrations [30]. In the kidney, the CaSR has different functions based on the tubular segments where it is located [31]. In brief, it is mainly involved in regulating the function of different tubular segments through modulating electrolyte and water excretion. Particularly, CaSR restrains passive and active reabsorption of calcium in distal tubules and enhances reabsorption of phosphate in proximal tubules [3]. Simultaneously, CaSR can increase excretion of proton and water in collecting ducts [27]. It is reported that the process of urolithiasis partly starts with an imbalance between excretion of water and insoluble stone-forming salts, leading to high concentrations that supersaturate urine and inner medullary collecting duct fluid [32]. Hence, CaSR plays an important role in urolithiasis and it is rational to hypothesize that its gene polymorphisms may be related to urolithiasis risk. An initial contribution was given by a study in knockout mice for the calcium channel TRPV5 [33]. Renkema and his colleagues found that transient receptor potential vanilloid 5 knockout (TPRV5−/−) mice lacked kidney stones despite urinary calcium (Ca^2+^) wasting and hyperphosphaturia and it perhaps resulted from their significant polyuria and urinary acidification. Activation of the renal CaSR promoted H^+^-ATPase-mediated H^+^ excretion and downregulation of aquaporin 2 (AQP 2), leading to urinary acidification and polyuria, respectively. The mice developed calcium-phosphate precipitate in collecting ducts only after inhibition of H^+^-ATPase activity that hampers urine acidification. Thus, we thought that CaSR polymorphisms might be involved in urolithiasis via influencing activities of CaSR gene and H-pump.

The incidence of gene polymorphisms can vary substantially among different racial populations. We therefore performed stratified analysis by ethnicity. Interestingly, contradictory associations were found in the A986S and R990G polymorphisms. For the A986S polymorphism, the SS genotype was associated with an increased urolithiasis risk in Asians but a decreased risk in Caucasians. Regarding the R990G polymorphism, the association between GG genotype and an increased risk of urolithiasis was only found in Caucasians. Even though the exact mechanism for the results was not well known, some concerns may account for it. Firstly, we presumed that the difference among ethnic groups might be a reflection of different genetic backgrounds and environmental context. Secondly, the sample size was relatively small, not having enough statistical power to explore the real association. Moreover, in view of diversity of possible comparisons and unavoidable flexibility of defining the correlations, associations may not necessarily reliable. For instance, selection bias, different matching criteria may play a role.

Different research objects may have an influence on the conclusions. We also conducted stratified analysis by subjects. Different subjects were categorized as PHPT patients, healthy population, and mixed population. Primary hyperparathyroidism is a disease characterized by excessive parathyroid cell proliferation and PTH secretion and occurs frequently in postmenopausal women [34, 35]. Kidney stones that are generally related to hypercalciuria are a common complication in PHPT patients [36]. Both Corbetta and Scillitani revealed that PHPT patients with the AGQ haplotype were susceptible to risk of urolithiasis [17, 18]. In our meta-analysis, we also found that PHPT patients who carried AA genotype were liable to stone formation. The calcium-sensing receptor (CaSR) regulates calcium homeostasis within a narrow physiological range by sensing extracellular calcium concentrations and by mediating alterations in PTH secretion and renal calcium reabsorption [37]. We speculated that the A986S polymorphism with a G-to-T mutation changed the activity of CaSR gene, which might further adjust PTH secretion, increase calcium clearance and affect calcium homeostasis. As a consequence, the risk of urolithiasis reduced. Nevertheless, regarding the R990G polymorphism, the results showed that there was no significant association between the G allele and urolithiasis risk in PHPT patients, which were not in accordance with the results from Corbetta and Scillitani. It was regrettable that we did not have adequate data and relevant studies to explain the inconformity, but relatively small sample size, selection bias, and ethnic difference could not be ignored.

It is worth noting that hypercalciuria, a disorder predisposing to calcium kidney stones, is vital in the mechanism of urolithiasis; therefore, we performed the first meta-analysis to evaluate the relationships between three CaSR polymorphisms and urine calcium concentration. The calcium-sensing receptor is the key controller of extracellular calcium homeostasis via its effects on regulation of parathyroid hormone secretion and renal calcium reabsorption [38]. Hypercalciuria is the most common abnormality identified in calcium stone formers seen in up to 40% of the stone formers [39]. So far, the associations between CaSR polymorphisms and urine calcium concentration were unclear. In our meta-analysis, we demonstrated the strong association between urine calcium concentration and the CaSR R990G polymorphism. A study from Vezzoli revealed that the extracellular calcium concentration producing the half-maximal intracellular calcium response was lower in HEK-293 cells transfected with the 990G allele than in those transfected with the wild-type allele. The G allele was recognized to cause a gain of CaSR function and increased susceptibility to hypercalciuria [20]. In the subgroup analysis, we merely discovered the Q1011E polymorphism had a linkage with low urine calcium concentration. However, we mentioned that there was heterogeneity among studies in overall comparisons and even subgroup analyses. We speculated that different methods to assess urine calcium could substantially influence the initial heterogeneity. We classified all eligible articles according to methods of measuring urine calcium and conducted a subgroup analysis. Heterogeneity was decreased after subgroup analysis, which confirmed our speculation. In the meanwhile, we noted that two studies from Vezzoli G might be the sources of heterogeneity. The degree of heterogeneity dramatically decreased after we dropped these two studies. We proposed some explanations although the exact reasons were not well known. Individuals included in this study had different genetic background and environmental factors. The sample size of each study varied and was relatively small. Besides, there were some other factors which might influence the urine calcium concentration, such as PH, phosphate, and PTH level.

Furthermore, despite the overall robust statistical evidence generated through this analysis, some limitations have been identified. Firstly, our results were based on unadjusted estimates, while a more precise analysis should be conducted if all individual raw data were available, which would allow for the adjustment by other covariates including age, sex, family history, and lifestyle. Secondly, only published studies which were retrievable in the selected databases were included in this meta-analysis, and some unpublished studies were missed. Thirdly, urolithiasis is a multifactorial disease that results from complex interactions between many genetic and environmental factors. It suggests that there will not be single gene or single environmental factor that has large effects on urolithiasis susceptibility. Fourth, heterogeneity could not be omitted because of methodological diversities between studies. Last but not least, this is the first meta-analysis regarding the comprehensive assessment of the relationship between CaSR polymorphisms with urolithiasis risk and urine calcium concentration. So numbers of published studies were not sufficiently large for a comprehensive analysis. The populations only come from Asians and Caucasians. Other ethnic populations should be involved in the future studies, such as Africans. Only four papers evaluated associations between the Q1011E polymorphism and urolithiasis risk; more studies should be conducted.

## 5. Conclusion, Future and Recommendations

Despite these limitations, the results of the present meta-analysis suggest that the G allele of CaSR R990G polymorphism increases susceptibility to urolithiasis and hypercalciuria. In other words, individuals that carry GG genotype have a higher risk of urolithiasis than those who carry RR genotype. The A986S and Q1011E polymorphisms were associated with urolithiasis and hypercalciuria in specific populations.

The identification of urolithiasis susceptible variants can provide new insight into its etiology. Moreover, it is an important step to individualize treatment and prevention programs. A well-established genetic marker surely would have a profound influence in screening and prediction of urolithiasis. To advance an understanding of the relationships between CaSR polymorphisms with urolithiasis risk and hypercalciuria, the following recommendations have been made. Firstly, try to decrease false positive and negative results by conducting the studies in a large sample with stratification by age, sex, food habit, lifestyle, and ethnicity. Secondly, more case-control studies or updated meta-analyses should be conducted to clarify the possible roles of CaSR polymorphisms in the etiology of urolithiasis. In addition, because the genetic background of stone formation is a complicated issue including single-candidate genes as well as epigenetic process, it is not simple to identify a single gene as an independent factor for urolithiasis. Combined effects of different gene polymorphisms need to be further analyzed.

## Figures and Tables

**Table 1:** Characteristics of studies included in the meta-analysis for associations between CaSR polymorphisms and urolithiasis risk.

CaSR A986S polymorphism							Case (*N*)			Control (*N*)			HWE
Year	Author	Ethnicity	Country	Genotyping	SOC	Subjects	A/A	A/S	S/S	A/A	A/S	S/S	
2013	Han et al. [15]	Asian	China	Sequencing	PB	PHPT and healthy	64	5	1	215	15	0	Y
2013	Han et al. [15]	Asian	China	Sequencing	HB	PHPT patients	64	5	1	88	6	0	Y
2011	Kim et al. [5]	Asian	Korea	PCR	PB	Healthy population	415	18	0	191	6	0	Y
2010	Shakhssalim et al. [13]	Asian	Iran	Sequencing	PB	Healthy population	71	26	2	93	14	0	Y
2009	Hamilton et al. [16]	Caucasian	Canada	Sequencing	PB	Healthy population	157	56	10	469	191	16	Y
2007	Scillitani et al. [17]	Caucasian	Canada	Sequencing	PB	PHPT and healthy	78	36	7	282	130	20	Y
2007	Scillitani et al. [17]	Caucasian	Canada	Sequencing	HB	PHPT patients	78	36	7	52	38	14	Y
2006	Corbetta et al. [18]	Caucasian	Italy	Taqman	HB	PHPT and healthy	30	12	1	91	42	4	Y
2006	Corbetta et al. [18]	Caucasian	Italy	Taqman	HB	PHPT patients	30	12	1	24	14	6	Y
2002	Vezzoli et al. [14]	Caucasian	Italy	Sequencing	PB	Healthy population	157	74		57	44		/

CaSR R990G polymorphism							Case (*N*)			Control (*N*)			HWE
Year	Author	Ethnicity	Country	Genotyping	SOC	Subjects	R/R	R/G	G/G	R/R	R/G	G/G	

2013	Han et al. [15]	Asian	China	Sequencing	PB	PHPT and healthy	20	40	10	50	119	61	Y
2013	Han et al. [15]	Asian	China	Sequencing	HB	PHPT patients	20	40	10	24	53	17	Y
2010	Shakhssalim et al. [13]	Asian	Iran	Sequencing	PB	Healthy population	87	10	2	105	2	0	Y
2009	Hamilton et al. [16]	Caucasian	Canada	Sequencing	PB	Healthy population	171	38	14	568	102	6	Y
2007	Scillitani et al. [17]	Caucasian	Canada	Sequencing	PB	PHPT and healthy	105	16	0	391	41	0	Y
2007	Scillitani et al. [17]	Caucasian	Canada	Sequencing	HB	PHPT patients	105	16	0	102	2	0	Y
2006	Corbetta et al. [18]	Caucasian	Italy	Taqman	HB	PHPT and healthy	34	9		128	9		/
2006	Corbetta et al. [18]	Caucasian	Italy	Taqman	HB	PHPT patients	34	9		43	1		/
2002	Vezzoli et al. [14]	Caucasian	Italy	Sequencing	PB	Healthy population	216	15		100	1		/

CaSR Q1011E polymorphism							Case (*N*)			Control (*N*)			HWE
Year	Author	Ethnicity	Country	Genotyping	SOC	Subjects	Q/Q	Q/E	E/E	Q/Q	Q/E	E/E	

2010	Shakhssalim et al. [13]	Asian	Iran	Sequencing	PB	Healthy population	94	5	0	107	0	0	Y
2007	Scillitani et al. [17]	Caucasian	Canada	Sequencing	PB	PHPT and healthy	113	8	0	402	30	0	Y
2007	Scillitani et al. [17]	Caucasian	Canada	Sequencing	HB	PHPT patients	113	8	0	102	2	0	Y
2002	Vezzoli et al. [14]	Caucasian	Italy	Sequencing	PB	Healthy population	222	9	0	98	3	0	Y

PB: Population-based study, HB: hospital-based study, and HWE: Hardy-Weinberg equilibrium.

**Table 2:** Characteristics of individual studies included in the meta-analysis of CaSR polymorphisms and urine calcium concentration.

CaSR A986S polymorphism											
Author	Year	Ethnicity	Country	Sample size	Subjects	AA	Mean	SD	AS + SS	Mean	SD
Han et al. [15]	2013	Asian	China	164	PHPT patients	151	413.47	204.96	13	506.05	180.5
Kim et al. [5]	2011	Asian	Korea	433	urolithiasis patients	415	190.3	112	18	138.6	84
Ferreira et al. [19]	2010	Mixed	Brazil	187	urolithiasis patients	177	239	100	10	280	86
Vezzoli et al. [20]	2007	Caucasian	Italy	243	Females	168	6.01	0.233	75	5.72	0.323
Pérez-Castrillón et al. [21]	2006	Caucasian	Spain	48	osteoporotic women	33	219	124	15	175	175
Corbetta et al. [18]	2006	Caucasian	Italy	79	PHPT patients	51	6.76	3.93	28	7.61	4.27
Harding et al. [22]	2006	Caucasian	England	188	dizygotic female twin pairs	141	0.44	0.15	47	0.46	0.18
Kelly et al. [23]	2006	Caucasian	England	121	Healthy populations	90	0.2	0.09	31	0.19	0.1
Vezzoli et al. [14]	2002	Caucasian	Italy	207	urolithiasis patients	133	6.91	0.26	74	7.96	0.46

CaSR R990G polymorphism											
Author	Year	Ethnicity	Country	Sample size	Subjects	RR	Mean	SD	RG + GG	Mean	SD

Corbetta et al. [18]	2006	Caucasian	Italy	79	PHPT patients	69	6.77	4.31	10	9.05	2.05
Harding et al. [22]	2006	Caucasian	England	188	Dizygotic female twin pairs	167	0.45	0.17	21	0.4	0.42
Vezzoli et al. [14]	2002	Caucasian	Italy	148	Urolithiasis patients	133	6.91	0.26	15	9.68	0.87
Shakhssalim et al. [13]	2010	Asian	Iran	206	Urolithiasis and healthy	192	187.06	99.75	14	214.43	99.23
Ferreira et al. [19]	2010	Mixed	Brazil	185	Urolithiasis patients	177	239	100	8	283	112
Vezzoli et al. [20]	2007	Caucasian	Italy	243	Females	220	5.82	0.196	23	7.59	0.63

CaSR Q1011E polymorphism											
Author	Year	Ethnicity	Country	Sample size	Subjects	QQ	Mean	SD	QE + EE	Mean	SD

Vezzoli et al. [20]	2007	Caucasian	Italy	243	Females	230	6.03	0.193	13	5.2	0.966
Harding et al. [22]	2006	Caucasian	England	188	Dizygotic female twin pairs	172	0.45	0.14	16	0.37	0.62
Vezzoli et al. [14]	2002	Caucasian	Italy	142	Urolithiasis patients	133	6.91	0.26	9	6	0.91
Shakhssalim et al. [13]	2010	Asian	Iran	206	Urolithiasis and healthy	201	233.6	139.9	5	187.81	98.73
Ferreira et al. [19]	2010	Mixed	Brazil	185	Urolithiasis patients	177	239	100	4	287	80

**Table:** (a) A986S

	*N* ^a^	AS versus AA	*P* ^b^	SS versus AA	*P* ^b^	SS versus AA/AS	*P* ^b^	*N* ^a^	AS/SS versus AA	*P* ^b^
Total	9	0.97 (0.79–1.19)	0.261	1.04 (0.48–2.25)	0.060	1.08 (0.53–2.19)	0.099	10	0.94 (0.71–1.25)	0.031
Ethnicity										
Asian	4	**1.64 (1.04–2.59)**	0.549	4.18 (0.84–20.86)	0.770	3.97 (0.80–19.76)	0.782	4	**1.78 (1.13–2.80)**	0.591
Caucasian	5	0.85 (0.67–1.07)	0.778	0.74 (0.31–1.80)	0.029	0.81 (0.35–1.85)	0.047	6	**0.78 (0.62–0.99)**	0.257
Subjects										
PHPT and healthy	3	0.98 (0.48–1.41)	0.918	1.35 (0.60–3.02)	0.412	1.35 (0.61–3.00)	0.421	3	1.03 (0.73–1.45)	0.773
PHPT patients	3	0.70 (0.44–1.10)	0.690	0.38 (0.10–1.42)	0.221	0.44 (0.12–1.44)	0.248	3	**0.62 (0.40–0.96)**	0.351
Healthy population	3	1.10 (0.82–1.47)	0.038	1.95 (0.91–4.18)	0.667	2.07 (0.95–4.52)	0.512	4	1.13 (0.65–1.96)	0.009
SOC										
PB	5	1.07 (0.84–1.36)	0.156	1.72 (0.97–3.05)	0.641	1.74 (0.99–3.06)	0.664	6	1.10 (0.77–1.57)	0.035
HB	4	0.74 (0.50–1.10)	0.809	0.40 (0.16–1.02)	0.330	0.44 (0.19–1.00)	0.376	4	**0.67 (0.46–0.96)**	0.442

**Table:** (b) R990G

	*N* ^a^	RG versus RR	*P* ^b^	GG versus RR	*P* ^b^	GG versus RR/RG	*P* ^b^	*N* ^a^	RG/GG versus RR	*P* ^b^
Total	6	1.45 (0.90–2.34)	0.029	1.90 (0.49–7.30)	0.000	1.84 (0.53–6.43)	0.000	9	**2.10 (1.23–3.58)**	0.001
Ethnicity										
Asian	3	1.27 (0.54–2.96)	0.059	0.63 (0.26–1.51)	0.213	0.65 (0.32–1.33)	0.246	3	1.26 (0.47–3.40)	0.017
Caucasian	3	1.74 (0.88–3.42)	0.063	7.01 (2.85–17.27)	0.745	6.73 (2.74–16.53)	0.717	6	**2.71 (1.50–4.91)**	0.044
Subjects										
PHPT and healthy	2	1.11 (0.65–1.90)	0.223	1.07 (0.07–15.53)	0.105	1.07 (0.09–12.75)	0.123	3	1.45 (0.61–3.41)	0.014
PHPT patients	2	2.40 (0.28–20.65)	0.009	0.76 (0.30–1.96)	0.575	0.79 (0.35–1.81)	0.646	3	3.61 (0.55–23.84)	0.004
Healthy population	2	2.28 (0.50–10.45)	0.051	7.57 (3.00–19.11)	0.877	7.27 (2.89–18.31)	0.851	3	**3.36 (1.00–11.28)**	0.064
SOC										
PB	4	1.31 (0.83–2.08)	0.123	2.87 (0.37–22.43)	0.000	2.83 (0.39–20.63)	0.000	5	1.64 (0.90–3.01)	0.013
HB	2	2.40 (0.28–20.65)	0.009	0.76 (0.30–1.96)	0.575	0.79 (0.35–1.81)	0.646	4	3.40 (0.97–11.85)	0.004

**Table:** (c) Q1011E

	*N* ^a^	QE versus QQ	*P* ^b^	EE versus QQ	*P* ^b^	EE versus QQ/QE	*P* ^b^	*N* ^a^	QE/EE versus QQ	*P* ^b^
Total	4	1.59 (0.91–2.81)	0.201	2.20 (0.40–12.07)	0.863	2.14 (0.39–11.75)	0.859	4	1.59 (0.91–2.81)	0.201

^a^Number of studies

^b^ *P* value of *Q* test for heterogeneity

SOC: Source of controls, HB: hospital-based control group, and PB: population-based control group.

**Table 4:** Summary of SMD and 95% CI for associations between urine calcium concentration and CaSR polymorphisms.

Polymorphism	Subgroup	*N* ^a^	Sample size	SMD (95% CI)	*P* value	*P* heterogeneity
A986S	All	9	1670	0.25 (−0.59–1.10)	0.56	<0.0001
	Caucasian populations	6	886	0.31 (−0.87–1.49)	0.605	<0.0001
	Asian populations	2	597	−0.02 (−0.92–0.88)	0.967	0.015
	Urolithiasis patients	3	827	1.00 (−1.32–3.32)	0.397	<0.0001
	Healthy populations	4	600	−0.35 (−1.00–0.29)	0.282	<0.0001
	PHPT patients	2	243	0.31 (−0.05–0.67)	0.093	0.511

R990G	All	6	1049	**2.52 (0.12**–**4.92)**	**0.039**	<0.0001
	Caucasian populations	4	658	3.62 (−0.18–7.42)	0.062	<0.0001
	Urolithiasis patients	2	333	4.00 (−3.00–10.99)	0.263	<0.0001
	Healthy populations	2	431	3.19 (−3.54–9.93)	0.353	<0.0001

Q1011E	All	5	964	−1.19 (−2.52–0.15)	0.081	<0.0001
	Caucasian populations	3	573	−**1.99 ** **(**−**3.74 to **−**0.24)**	**0.026**	<0.0001
	Urolithiasis patients	2	327	−1.14 (−4.29–2.01)	0.477	<0.0001
	Healthy populations	2	431	−1.63 (−4.11–0.86)	0.201	<0.0001

^a^The number of studies.
